# The capacity for generating cognitive reappraisals is reflected in asymmetric activation of frontal brain regions

**DOI:** 10.1007/s11682-016-9537-2

**Published:** 2016-03-02

**Authors:** Ilona Papousek, Elisabeth M. Weiss, Corinna M. Perchtold, Hannelore Weber, Vera Loureiro de Assunção, Günter Schulter, Helmut K. Lackner, Andreas Fink

**Affiliations:** 1 Department of Psychology, Biological Psychology Unit, University of Graz, Graz, Austria; 2 Department of Psychology, University of Greifswald, Greifswald, Germany; 3 Institute of Physiology, Medical University of Graz, Graz, Austria

**Keywords:** Cognitive reappraisal, Maximum performance, EEG asymmetry, Prefrontal cortex

## Abstract

Encouraging patients to use cognitive reappraisal constitutes the core of modern psychotherapeutic approaches. However, evidence for specific neural correlates of the capacity for cognitive reappraisal, which is a necessary prerequisite for the effective implementation of cognitive reappraisal in everyday life, has been sparse to date. In the present study, the capacity for cognitive reappraisal was studied in terms of the participants’ inventiveness in generating alternative appraisals of anger-evoking events, and was correlated with frontal EEG alpha asymmetry recorded while the participants were generating reappraisals as well as during a common creative idea generation task. During cognitive reappraisal efforts, individuals higher on the capacity for generating cognitive reappraisals showed more left-lateralized activity in lateral prefrontal cortex, specifically in ventrolateral prefrontal cortex extending toward the frontal pole. This effect was observed independently from the activation during novel idea generation without emotional component, indicating that specific demands are implicated in the generation of reappraisals of emotional events. Taken together, the results indicate that individuals higher on the capacity for cognitive reappraisal are more capable or more prone to recruit appropriate brain regions when the situation demands coming up with alternative appraisals of stressful events. The findings may stimulate the development of more individually targeted interventions.

## Background

Starting from seminal work of Lazarus and others (see Lazarus 1993 for review), the view that an individual’s appraisal of a stressful situation (in terms of meaning and significance), rather than the situation itself, determines the quality and intensity of the emotional response has gained wide acceptance (see also Ellsworth and Scherer 2003). Cognitive reappraisal refers to deliberately viewing an emotionally evocative event from a different perspective and re-interpreting its meaning, thereby changing its emotional impact (Lazarus and Alfert 1964; Lazarus and Folkman 1984). It is regarded a particularly effective strategy in coping with adverse events (e.g., Augustine and Hemenover 2009; Webb et al. 2012). Encouraging patients to use cognitive reappraisal constitutes the core of modern psychotherapeutic approaches. With extensive practice, that is, with frequent repetition in the same context, cognitive reappraisal may eventually become a habitual response to situations involving stress or negative mood (Hertel 2004), which is thought to have positive implications for psychological health and well-being in the long run (e.g., Garnefski et al. 2002; Gross and John 2003). Yet, effective therapeutic interventions aiming at increasing the patients’ use of cognitive reappraisal may also require that patients improve their capability to implement this strategy.

To date, studies specifically focusing on neural correlates of the capability for cognitive reappraisal are limited. Several studies examined which brain structures were active while participants were making deliberate efforts to reappraise negative stimuli (mostly pictures), in order to reduce their emotional impact. Besides the medial frontal cortex, which most likely can be considered as a relay station between circuits involved in reappraising the emotional significance of stimuli and subcortical circuits crucial for the generation of emotional responses (including the amygdala; Johnstone et al. 2007), reappraisal efforts most consistently increased activation in lateral prefrontal cortex, especially in the left hemisphere (Dillon and Pizzagalli 2013; Kalisch 2009; Ochsner et al. 2002, 2012; Phan et al. 2005). This seems to be particularly true in earlier stages of experimental reappraisal, which are presumably dominated by efforts to generate alternative appraisals, compared to later periods that are more dominated by maintenance processes (Kalisch 2009). However, there has also been some heterogeneity in findings (e.g., Kohn et al. 2014), which may in part be due to the difficulty in typical cognitive reappraisal tasks to ascertain that participants are actually compliant when asked to use this specific strategy. Targeted studies showed that a large proportion of participants did not strictly adhere to which emotion regulation strategy they were instructed to use during watching negative affective material (Demaree et al. 2006). In addition, studies have typically used a no-regulation (just watch) condition as a reference, whereby activations in many brain regions may occur that are not specifically due to cognitive reappraisal but, for instance, to the mere presence of cognitive effort (Phan et al. 2005).

Compared to studies examining brain activations after participants were instructed to use reappraisal during watching negative stimuli, evidence for specific neural correlates of the capacity for cognitive reappraisal has been sparse to date. There is, nevertheless, some first evidence suggesting that the capacity for cognitive reappraisal may be related to the degree to which relevant brain regions are activated during reappraisal efforts. Depressed patients, who have difficulties in cognitively reappraising negative emotional events (Beauregard et al. 2006), showed attenuated prefrontal activation, and depression severity was inversely correlated with the modulation of activation in left prefrontal cortex regions during instructed reappraisal (Dillon and Pizzagalli 2013; Johnstone et al. 2007; Townsend et al. 2013). Similarly, older people showed attenuated activation in the left prefrontal cortex during a reappraisal task and at the same time were less successful in using reappraisal in terms of decreasing negative affect (Opitz et al. 2012).

In the present study, we used a more specific, novel approach of investigating neural correlates of the capacity for cognitive reappraisal by studying individuals’ reappraisal inventiveness, which refers to the specific capability of generating possible alternative appraisals of self-relevant negative emotional events. In a more indirect approach, some researchers had considered the effectiveness of reappraisal during a laboratory challenge, that is, an individual’s regulation success in terms of differences in emotion intensity between an experimental condition in which they were asked to use reappraisal and a no-regulation condition as an indicator of reappraisal ability (e.g., Lee et al. 2012; Mauss et al. 2013; McRae et al. 2012; Troy et al. 2010, 2013). But rather being an indicator of the outcome of what people typically do, this approach does not conform to an ability test in a narrower sense (as used in psychometrics), in which maximum performance is the variable of interest - referring to what people can do at their best (Cronbach 1970; see also Malooly et al. 2013). The present study focuses on a different concept of reappraisal ability by exploring the capacity to ad hoc generate many different reappraisals for a critical situation. While widely used in psychology (e.g., intelligence tests) and several medical disciplines such as neurology (e.g., motor performance tests) and psychiatry (e.g., neuropsychological testing), the use of maximum performance measures, capturing what individuals are theoretically capable of doing, is a novel concept in psychotherapy research. The theoretical capacity for generating cognitive reappraisals can be regarded a direct prerequisite for the ability to effectively implement cognitive reappraisal in everyday life (Weber et al. 2014).

We studied neural correlates of an individual’s capacity for generating reappraisals in the context of a research tradition that has recurrently demonstrated correlations between dispositional as well as transient changes of lateral asymmetry in the prefrontal cortex and relevant constructs in the affective domain. According to the premises of this approach, the left and right prefrontal cortical hemispheres are differentially involved in processes modulating affective responses to emotional challenges. More specifically, it is assumed that relatively greater left than right prefrontal activity, measured by EEG alpha asymmetry, is associated with the ability to modulate emotional responses and, hence, with more adaptive responses to emotionally challenging events (for review see Davidson 1998; Harmon-Jones et al. 2010).

First important evidence for the relevance of hemispheric asymmetry in the context of the present study’s specific research aims was provided by the study of Johnstone et al. (2007) who demonstrated left-lateralized activation in the ventrolateral prefrontal cortex during a cognitive reappraisal task in healthy individuals, whereas patients with depression showed bilateral prefrontal activation. The authors concluded that the absence of left-lateralized activation and the increased activation in right prefrontal cortex indicated an inappropriate or inefficient engagement of prefrontal regulatory circuitry, which may be linked to the difficulties of depressed patients to adequately cope with adverse events. More generally, several empirical studies indicated functional deficits, if a brain region on which these functions depend was inadequately activated, and that lateralized activation of specific relevant brain regions was associated with better performance on hemisphere specific cognitive tasks (Davidson et al. 1990; Gur et al. 1994, 2000; Gur and Reivich 1980; Papousek et al. 2011; Papousek and Schulter 2004; Wendt and Risberg 1994).

According to the capability model of frontal EEG asymmetry (Coan et al. 2006), inter-individual differences in prefrontal EEG alpha asymmetry responses are produced by the interaction between the emotional demands of specific situations and the capability or proneness of the individual to recruit appropriate brain regions in that particular context. In line with this idea, several studies showed that associations with individual differences variables were more prominent or only became apparent when prefrontal EEG alpha asymmetry was recorded in directly relevant situational contexts (Coan et al. 2006; Crost et al. 2008; Goodman et al. 2013; Papousek et al. 2013a, 2014; Perez-Edgar et al. 2013; Stewart et al. 2014; Wacker et al. 2013). Therefore, in the present study the participants’ capacity for cognitive reappraisal was correlated with the EEG alpha asymmetry recorded directly while they were generating reappraisals. We used the Reappraisal Inventiveness Test (Weber et al. 2014), in which participants are instructed to imagine given anger-eliciting situations happening to them and to generate as many different ways as possible to reappraise the situation in a way that diminishes their anger. Participants’ responses to these items were recorded and were used for the assessment of the participants’ capacity for generating cognitive reappraisals.

The generation of reappraisals may at least in part rely on basic executive functions such as the inhibition of highly activated or prepotent representations, memory updating, and cognitive switching (Joormann and Gotlib 2010; Malooly et al. 2013; Weber et al. 2014). In line with this, research suggested that an individual’s executive functioning determines the success of using cognitive reappraisal in terms of reducing negative affect (Pe et al. 2013a, b; Schmeichel et al. 2008). However, not studying reappraisal ability as such (in terms of maximum performance), the latter findings only indirectly indicated a relationship between executive functions and the capacity for cognitive reappraisal in a narrower sense.

Executive functions are generally known to be associated with the integrity of the frontal lobes (e.g., Gazzaley and D’Esposito 2007). If studies were also concerned with hemispheric asymmetry, activation during relevant cognitive processes was typically observed in left lateral prefrontal regions (Badre and Wagner 2007; Hirshorn and Thompson-Schill 2006; Jahanshahi et al. 2000; Jonides and Nee 2006; Jonides et al. 1998; Joppich et al. 2004; Oztekin et al. 2009). Some of the executive processes putatively required for generating reappraisals of emotionally laden situations also play a major role in the generation of novel (creative) ideas to given open problems (Beaty and Silvia 2012; Benedek et al. 2012; Fink and Benedek 2014; Gilhooly et al. 2007; Runco 2010). Left-lateralized activation in prefrontal cortex has also been observed during creative idea generation (Benedek et al. 2014; Fink et al. 2009). However, in cognitive reappraisal, which per definition refers to processing emotionally relevant information and changing its emotional impact, additional or more specific demands may be present, which may also be reflected at the level of the brain.

Therefore, in order to examine whether specific activation is relevant in the context of generating cognitive reappraisals compared to novel idea generation without emotional component, in the present study EEG alpha asymmetry was also recorded during a common creative idea generation task, which required participants to think of as many different creative uses for conventional objects (such as a brick or a barrel) as possible. Thereby, it could be tested whether individual differences in the capacity for cognitive reappraisal were explained by unique variance of EEG alpha asymmetry during the generation of reappraisals, that is, by variance that was independent from activations during the novel idea generation without emotional component.

## Methods

### Participants

The final sample comprised 78 right-handed female university students of different faculties, aged between 18 and 35 years (*M* = 22.4, *SD* = 3.4). A student sample was chosen, because the used version of the behavioral test of the capacity for generating cognitive reappraisals (Weber et al. 2014) had been tailored for a student population (the items include negative experiences that students can easily imagine happening to them). Individuals who reported having a neuropsychiatric disease or using psychoactive medication were not included in the study. Moreover, only participants who had a minimum of 30 s of artifact free EEG data in each of the experimental conditions at each of the used electrodes were included in the sample. Handedness was assessed by a standardized hand skill test (Hand Dominance Test; Papousek and Schulter 1999; Steingrüber and Lienert 1971). A female-only sample was chosen in order to avoid any confounding effects produced by potential gender differences in emotion-related abilities or typical behavior (e.g., Domes et al. 2010; Freudenthaler and Papousek 2013), and because women may be more motivated to down-regulate anger than men for social reasons (Evers et al. 2005). Depression scores (Center of Epidemiological Studies Depression scale; CES-D; German version by Hautzinger and Bailer 1993) ranged from 1 to 31 (*M* = 11.4, *SD* = 6.6; maximum possible score = 60). Participants were requested to refrain from alcohol for 12 h and from coffee and other stimulating beverages for 2 h prior to their lab appointment, and to come to the session well rested.

### Tasks

#### Generation of cognitive reappraisals

**Fig. 1:**
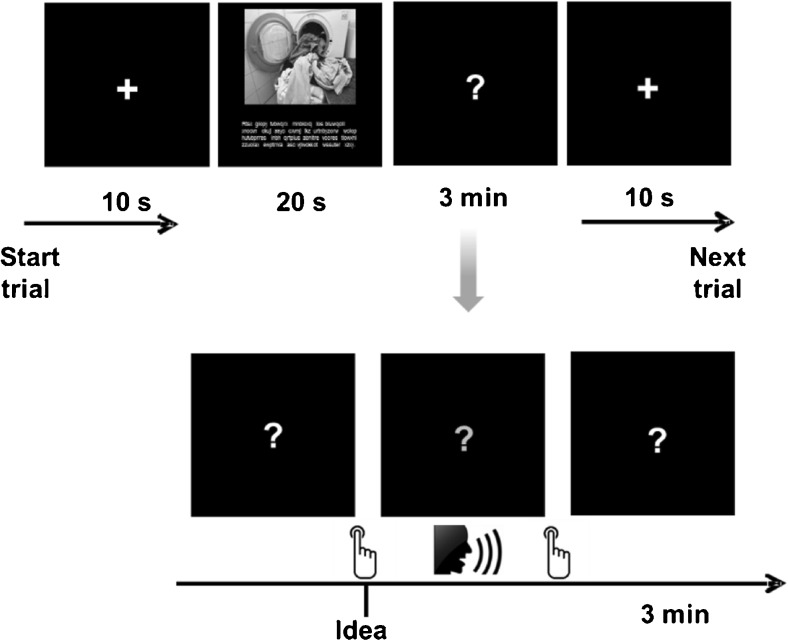
Schematic representation of cognitive reappraisal task

The Reappraisal Inventiveness Test (RIT; Weber et al. 2014) consists of anger-eliciting vignettes that, in line with cognitive emotion theories, depict the behavior of another person who willingly or carelessly induces harm. Each vignette is supplemented by a matching photograph to make the situation more vivid. Participants are instructed to imagine the situation happening to them and to generate and write down as many different ways as possible to think about or appraise the situation in a way that diminishes anger. In the present study, the items of the Reappraisal Inventiveness Test were slightly adapted to make them suitable for concurrent EEG recording and later analysis: Each vignette was presented on a computer screen for 20 s. (The presentation time corresponded to the time scheduled in the original test for reading and imagining the vignettes. Pre-tests had ensured that the time-frame provided enough time for reading the vignette and was short enough for the production of reappraisals to only begin during the stimulus-free recording interval afterwards). Participants were instructed to imagine the situation happening to them and to generate as many different ways as possible to think about or appraise the situation in a way that diminishes anger. They were instructed to press a button whenever a new appraisal came into their mind, and to vocalize the idea concisely in one or two short sentences immediately after pressing the button. Then they were asked to press the button again, and the task was resumed until the allotted time of 3 min had elapsed. In doing so, we were able to separate EEG segments related to the generation of reappraisals from segments contaminated with the production of speech. This protocol has proved to be eminently suitable in previous relevant research in the creativity domain (Fink et al. 2007). The allotted time of 3 min for each item corresponded to the original procedure of the Reappraisal Inventiveness Test. Participants’ vocal responses were audiotaped for later analysis, and adherence to the protocol was carefully monitored. See Fig. 1 for a schematic representation of one item of the cognitive reappraisal task.

The protocol comprised eight vignettes, the four original vignettes of the Reappraisal Inventiveness Test (Weber et al. 2014), and four additional vignettes that were pre-tested in pilot tests in order to select items matching the main characteristics of the original vignettes to the best possible extent. On average, participants of the final study sample generated *M* = 4.5 valid (distinguishable) reappraisals (*SD* = 1.8) to each of the original vignettes and *M* = 4.2 (*SD* = 1.5) to each of the additional ones. Number of categorically different reappraisals per item (see Weber et al. 2014) was *M* = 3.1 (*SD* = 0.9) and *M* = 3.0 (*SD* = 1.0), respectively. In ratings in which the participants rated for each vignette the anger that they would experience when confronted with the situations depicted in the vignettes (rated after completion of all items, vignettes were shown again; 7-point scales ranging from 0 ‘not angry at all’ to 6 ‘extremely angry’), ratings were *M* = 4.0 (*SD* = 1.6), *M* = 4.2 (*SD* = 1.4), *M* = 4.6 (*SD* = 1.5), *M* = 3.6 (*SD* = 1.5) for the original and *M* = 4.3 (*SD* = 1.2), *M* = 4.7 (*SD* = 1.4), *M* = 2.8 (*SD* = 1.4), *M* = 2.9 (*SD* = 1.7) for the additional vignettes. In one-sample t-tests anger ratings of all eight vignettes differed significantly from zero (*t*-values ranging from 15.1 to 30.3, all *p*-values < .001), indicating that all depicted situations were indeed perceived as anger evoking.

#### Generation of novel ideas without emotional component

**Fig. 2:**
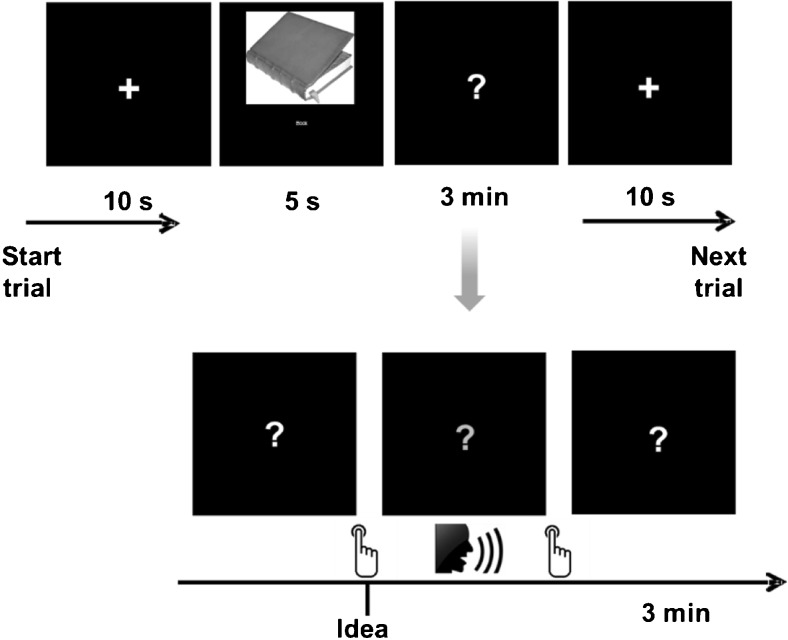
Schematic representation of creative idea generation task

The Alternate Uses Task, which is one of the most commonly applied creativity tasks (Fink et al. 2007; Wilson et al. 1953) requires participants to think of many different creative uses for conventional objects such as a brick or a barrel. Eight items were selected from previous studies (e.g., Fink et al. 2012), in order to match them to the reappraisal tasks with respect to the average number of generated ideas. Total numbers of generated ideas in the study sample were *M* = 46.8 (*SD* = 20.6) for the cognitive reappraisal task and *M* = 50.2 (*SD* = 21.1) for the alternate uses task (*t*(77) = 1.8, ns.). Furthermore, the stimulus words were presented conjointly with images of the respective objects, in order to make the task as comparable as possible to the cognitive reappraisal task. Participants were instructed to generate as many and as original creative uses for the objects as possible, and to press a button whenever they became aware of an idea. Analogous to the cognitive reappraisal task, they were asked to vocalize the idea immediately after pressing the button, after which the task was resumed until the allotted time of 3 min had elapsed (see also Fink et al. 2007). See Fig. 2 for a schematic representation of one item of the creative idea generation task.

### Capacity for cognitive reappraisal

Participants’ responses to the Reappraisal Inventiveness Test were used for the assessment of their capacity for generating cognitive reappraisals. In line with the scoring procedure of the Reappraisal Inventiveness Test (Weber et al. 2014), two scales were used: RIT-fluency refers to the total number of generated non-identical ideas that qualified as cognitive reappraisals. RIT-flexibility refers to the number of categorically different reappraisals (for the category scheme see Weber et al. 2014). Assessing what people can do at their best, the scales conform to the definition of ability (“maximum performance”) tests (Cronbach 1970). The scores obtained for each vignette were aggregated to obtain the scores for RIT-fluency and RIT-flexibility. In the present study, all responses were independently rated by two experimenters. Inter-rater reliabilities were *ICC* = .93 for RIT-fluency and *ICC* = .93 for RIT-flexibility, respectively. Mean RIT-fluency scores (sum over all eight items) were *M* = 34.8 (*SD* = 12.6). Mean RIT-flexibility scores were *M* = 24.5 (*SD* = 7.0). Internal consistency reliabilities (Cronbach’s alpha) were α = .93 (RIT-fluency) and α = .89 (RIT-flexibility). The intercorrelation between the two scales was *r* = .87.

### EEG recording and quantification

EEG was recorded using a Brainvision BrainAmp Research Amplifier (Brain Products) and a stretchable electrode cap, referenced to the nose and re-referenced offline to a mathematically averaged ears reference (Hagemann 2004). Impedance was kept below 5 kΩ for all electrodes. EOG measures were obtained for identification of ocular artifacts. The vertical EOG was recorded from the supra- and sub-orbit of the right eye, the horizontal EOG was recorded from the outer canthi using adhesive Ag/AgCl electrodes. All data were inspected visually, in order to eliminate intervals in which ocular or muscle artifacts occurred. For the assessment of EEG asymmetry during the cognitive reappraisal task and the creative idea generation task, only the time frames in which participants were mentally generating ideas were used, that is, reading and speaking intervals were excluded. Power spectra (epoch length 1 s, overlapping 50 %, Hanning window) were averaged across all artifact-free intervals for an individual. Following the common approach in the field, power within the alpha frequency band (8–12 Hz) was used for the analyses. Laterality coefficients (LC) were computed for each electrode pair as LC = ((R−L)/(R + L)) × 100, where R denotes the electrode over the right hemisphere and L denotes the homologous electrode over the left hemisphere. The calculation of LC has a long tradition in laterality research, because it separates the variance in asymmetry from the variance in general magnitude (e.g., Porac and Coren 1981). In EEG studies, this asymmetry ratio is equivalent to another common metric (lnR - lnL), with which it is virtually perfectly correlated (Davidson 1988; Papousek and Schulter 2002). However, LC allows easier comparison of data from different studies, different frequency bands, and locations (Pivik et al. 1993), and has been used in numerous EEG studies in relevant research contexts (e.g., Papousek et al. 2011, 2012, 2013a, b, 2014; Papousek and Schulter 2004). Following the common approach in EEG alpha asymmetry research, we interpret relatively lower alpha power in one hemisphere than the other as relatively greater cortical activity in this hemisphere (see, Allen et al. 2004 for a review of evidence and Harmon-Jones 2006; Michels et al. 2010; Scheeringa et al. 2011 for recent experimental research supporting the assumption that EEG alpha band activity obtained in time frames of several seconds or minutes is inversely related to cortical activity). Consequently, positive values of LC, indexing higher alpha activity in the right than in the left hemisphere, indicate relatively greater left than right hemisphere cortical activity.

### Procedure

After completing the handedness test, participants were seated in an acoustically and electrically shielded examination room, and electrodes were attached. EEG was recorded in an initial two minutes rest period with closed eyes. Participants were then instructed for the first task and were given a practice item. After completing the first task block, they were instructed for the second task (including a practice item), and completed the second task block. Order of tasks (cognitive reappraisal task, creative idea generation task) was counterbalanced. Following the reappraisal task block, participants completed the anger ratings for each of the depicted situations using the computer mouse. After the tasks, electrodes were detached and the participants were given the opportunity to wash and dry their hair. Finally, they completed the depression questionnaire.^1^ During the EEG recordings, the experimenters were outside the examination room, and participants were carefully monitored through a camera and an intercom.

### Statistical analysis

Participants’ capacity for cognitive reappraisal was correlated with prefrontal EEG alpha asymmetry during the generation of reappraisals in standard multiple regression analyses using alpha asymmetry during the generation of reappraisals and alpha asymmetry during the creative idea generation task as predictors and performance on the Reappraisal Inventiveness Test (either RIT-fluency or RIT-flexibility) as the dependent variable. This approach allowed to examine if individual differences in the capacity for cognitive reappraisal were explained by variance of cortical asymmetry during the generation of reappraisals that included overlapping variance of asymmetry during creative idea generation (zero-order correlations); as well as whether individual differences in the capacity for cognitive reappraisal were also explained by unique variance of EEG alpha asymmetry during the generation of reappraisals that was independent from variance of asymmetry during novel idea generation without emotional component (semipartial correlations). Both was relevant to the present research aims: The two tasks may share important executive requirements and, therefore, also major parts of their neurological substrates, hence the capacity for generating cognitive reappraisals may be correlated with cortical activation patterns that are present during both tasks. At the same time additional demands may be present in cognitive reappraisal as compared to novel idea generation without emotional components, which may produce correlations between the capacity for generating cognitive reappraisals and unique activations during the cognitive reappraisal task. The regression analyses were performed for each of the three prefrontal electrode pairs: ventrolateral prefrontal (F7, F8), frontopolar (Fp1, Fp2), dorsolateral prefrontal (F3, F4). According to the specific research background and hypotheses of the present study, we focused on the prefrontal electrode pairs in our analyses, in order to avoid an unnecessary great number of statistical analyses. As a supplemental analysis, average effects of the task conditions were examined by means of a oneway multivariate analysis of variance using condition (rest, generation of cognitive reappraisals, generation of novel ideas without emotional component) as the within-subjects factor and EEG alpha asymmetry at the three prefrontal electrode pairs as the dependent variables. A significance level of *p* < .05 (two-tailed) was used for all analyses.

## Results

### Capacity for cognitive reappraisal

**Fig. 3:**
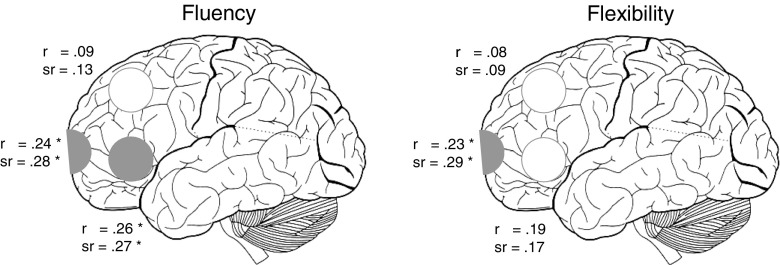
Prediction of the capacity for cognitive reappraisal (fluency and flexibility scales of the Reappraisal Inventiveness Test) by prefrontal EEG alpha asymmetry (LC) during the generation of reappraisals. *r* … zero-order correlations, *sr* … semipartial correlations controlling for EEG alpha asymmetry during novel idea generation without emotional component, * *p* < .05 (*r* = .26, *p* = .024; *sr* = .27, *p* = .016; *r* = .24, *p* = .038; *sr* = .28, *p* = .015; *r* = .23, *p* = .042; *r* = .29, *p* = .010). Note the high inter-correlation between the fluency and the flexibility aspect of the capacity for generating cognitive reappraisals (*r* = .87)

In the analyses examining prefrontal EEG alpha asymmetry (LC) during the experimental tasks, asymmetry at the ventrolateral prefrontal (*F*(2,75) = 3.6, *p* = .033) and the frontopolar electrodes (*F*(2,75) = 3.2, *p* = .048) predicted the participants’ fluency in generating cognitive reappraisals (RIT-fluency), but not EEG alpha asymmetry at the dorsolateral prefrontal electrodes (*F*(2,75) = 0.7, ns.). Zero-order and semi-partial correlations between prefrontal EEG alpha asymmetry during the generation of reappraisals and participants’ capacity for cognitive reappraisal are shown in Fig. 3. They indicate that primarily unique variance of EEG alpha asymmetry during the generation of reappraisals that was independent from variance of asymmetry during novel idea generation without emotional component accounted for the correlations with the capacity for cognitive reappraisal. The flexibility component of the capacity for generating cognitive reappraisals (RIT-flexibility) was only predicted by EEG alpha asymmetry at the frontopolar electrodes (*F*(2,75) = 3.5, *p* = .035; ventrolateral prefrontal: *F*(2,75) = 1.7, ns.; dorsolateral prefrontal: *F*(2,75) = 0.3, ns.; Fig. 3). EEG alpha asymmetry during the generation of novel ideas without emotional component did not show significant correlations with RIT-fluency or RIT-flexibility (*r*’s ranging from .01 to .11). Please see Table 1 for details of the regression analyses.^2^

**Table 1:** Details of multiple regression analyses

	Generation of				
	Cognitive reappraisals		Novel ideas w/o emotional component		
	β (p)	sr^2^	β (p)	sr^2^	R (p)
RIT-fluency					
LC ventrolateral prefrontal	.45 (.016)	.07	−.24 (.186)	.02	.30 (.033)
LC frontopolar	.36 (.015)	.08	−.20 (.183)	.02	.28 (.048)
LC dorsolateral prefrontal	.22 (.251)	.02	−.17 (.375)	.01	.13 (.514)
RIT-flexibility					
LC ventrolateral prefrontal	.29 (.127)	.03	−.12 (.529)	.01	.21 (.195)
LC frontopolar	.38 (.010)	.09	−.24 (.107)	.03	.29 (.035)
LC dorsolateral prefrontal	.16 (.429)	.01	−.10 (.617)	.00	.10 (.710)

Analogous multiple regression analyses with asymmetry at more posterior electrode pairs as predictors (C3/C4, T3/T4, P3/P4, P7/P8, O1/O2) did not yield any significant results (all *F* ≤ 1.5).

### Average effects of task conditions

**Fig. 4:**
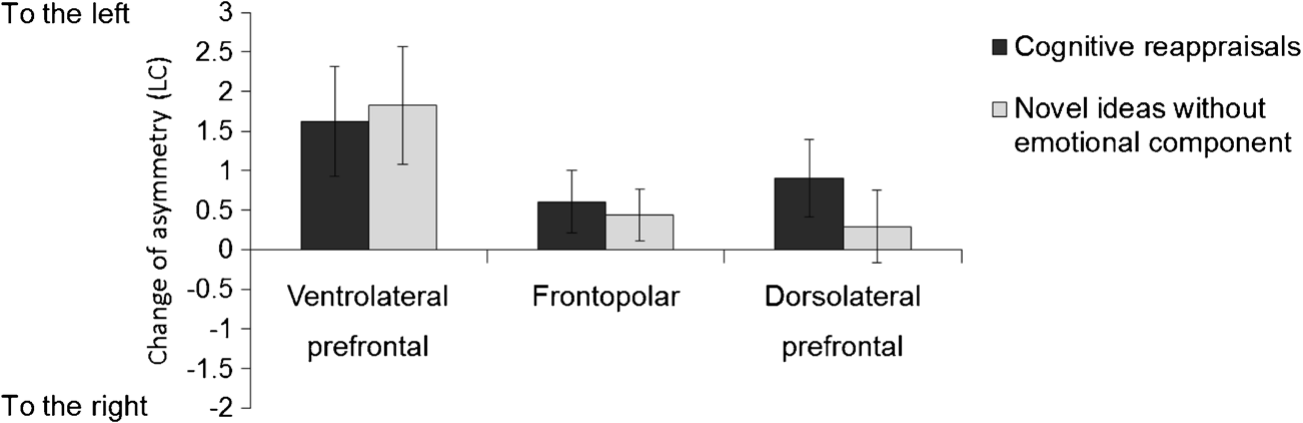
Average effects of task conditions on prefrontal EEG alpha asymmetry (LC). Relative changes (task minus rest)

In the analysis of variance testing the average effects of the task conditions on EEG alpha asymmetry (LC), the multivariate main effect of task was significant (*F*(6,306) = 2.3, *p* = .035, η^2^ = .04). Univariate comparisons indicated relatively greater left-lateralized asymmetry during the tasks compared to the resting condition at the ventrolateral prefrontal electrodes (*F*(2,154) = 4.3, *p* = .015, η^2^ = .05; frontopolar: *F*(2,154) = 1.7, ns.; dorsolateral prefrontal: *F*(2,154) = 2.3, ns.). To specifically compare the two task conditions, paired t-Tests were additionally computed, which indicated relatively greater left-lateralized asymmetry during the cognitive reappraisal task compared to the creative idea generation task at the dorsolateral prefrontal electrodes, which was significant at trend level (*t*(77) = 1.9, *p* = .062; ventrolateral prefrontal: (*t*(77) = 0.3, ns.; frontopolar electrodes: *t*(77) = 0.6, ns.). Figure 4 shows the mean values for the two tasks relative to the EEG alpha asymmetry in the resting condition.

## Discussion

The main finding of the present study is that individuals showing greater capacity for cognitive reappraisal in terms of greater inventiveness in generating reappraisals displayed more relative left-lateralized activity in lateral prefrontal cortex while they were attempting to generate reappraisals, specifically in ventral regions extending toward the frontal pole. The demonstration of a relationship between the capacity for generating cognitive reappraisals and lateralized activity in prefrontal cortex during the explicit generation of reappraisals is novel. Nevertheless, the finding can well be integrated into the existing literature. Functional magnetic resonance imaging (fMRI) studies investigating brain activation patterns after participants were instructed to use reappraisal to reduce the negative impact of emotional pictures consistently showed increased activation in left lateral prefrontal cortex, particularly at earlier periods of the experimental reappraisal phases that were presumably dominated by efforts to generate alternative appraisals (compared to later periods that were more dominated by maintenance processes; Dillon and Pizzagalli 2013; Kalisch 2009; Ochsner et al. 2002; Phan et al. 2005). At the same time, activation in multiple other cortical regions was typically observed in fMRI studies in which participants were instructed to reappraise emotional picture content, but obviously not all of these activations were linked to processes specifically implicated in cognitive reappraisal (Phan et al. 2005).

Using more specific experimental paradigms, recent studies strongly supported the particular importance of ventral and rostral regions of the left prefrontal cortex for cognitive reappraisal. Compared to other strategies such as expressive suppression and distraction, cognitive reappraisal efforts uniquely activated left ventrolateral prefrontal and orbitofrontal cortex, whereas increased activation in other cortical regions was observed during the other strategies (Dörfel et al. 2014; Price et al. 2013). Jensen et al. (2012) reported increased activation in the left ventrolateral prefrontal cortex extending to rostral portions during a pain stimulus in patients with chronic pain after they had received cognitive-behavioral therapy, compared to a control group without psychological treatment. The neuronal effect was correlated with reductions in anxiety but not with ratings of pain intensity. The authors, therefore, suggested that the observed increased activation of the left ventrolateral prefrontal cortex was not related to direct inhibition of pain, but to improved cognitive reappraisal of the experience of pain, through increased access to executive cognitive functions of the prefrontal cortex. In a similar vein, decreased pain catastrophizing after cognitive-behavioral therapy in patients with chronic pain was associated with increased gray matter volume in left lateral prefrontal cortices (Seminowicz et al. 2013). Behavioral experimental evidence confirmed the trainability of cognitive reappraisal (Denny and Ochsner 2014). However, none of the fMRI studies so far have related the capacity for cognitive reappraisal (in terms of maximum performance) to brain activations during the generation of alternative appraisals, and none have specifically focussed on hemispheric differences.

In the present study, neural correlates of the capacity for generating cognitive reappraisals were studied following a research tradition that specifically focuses on the relative difference in activation between the hemispheres. In this approach, it is assumed that in some cases the relative difference in activation between the hemispheres is more important than the absolute level of independent left or right hemisphere activation, particularly when studied brain processes are lateralized (as it seems to be the case with cognitive reappraisal). That is, in some instances, no effect of increased left-hemisphere activation may be observed if at the same time the right hemisphere is also more activated. This seems to apply to many cases in the emotional domain (Harmon-Jones 2006; Heller et al. 1997), but can also be observed in the cognitive domain, when hemisphere-specific performances are studied (Davidson et al. 1990; Gur et al. 1994). Accordingly, studies often failed to detect effects when the absolute activity at individual sites was examined and data of the left and right hemisphere were not related to each other by using appropriate laterality coefficients (e.g., Blackhart and Kline 2005; Cole et al. 2012; Harmon-Jones 2006; Papousek and Schulter 2004; Shankman et al. 2011). While in EEG alpha asymmetry research, effects of cognitive reappraisal have not yet been specifically examined, the present study adds to the evidence supporting the importance of hemispheric differences in the context of coping with negative events (Blackhart and Kline 2005; Davidson 2004a; Jackson et al. 2003; Nusslock et al. 2011). Moreover, the findings may be related to previous evidence suggesting an association between left-lateralized prefrontal EEG alpha asymmetry, in particular at the ventrolateral prefrontal electrodes, and affective flexibility (Papousek et al. 2012, 2013b, 2014), which implies the selection of appropriate and the inhibition of inappropriate processes (Thayer and Friedman 2002). Finally, the present results are nicely in line with the proposition of Johnstone et al. (2007) that the absence of left-lateralized activation in the ventrolateral prefrontal cortex and concurrent enhanced activation in right prefrontal cortex, which were observed in depressed patients during reappraisal efforts, reflect an inefficient engagement of prefrontal regulatory circuitry. Similar associations between the failure to generate appropriate asymmetry of activation during the execution of tasks and poorer task performance have been found in other cognitive domains in EEG asymmetry studies (e.g., Papousek and Schulter 2004).

The involvement of ventral prefrontal cortex strongly supports the notion that executive functions play a major role in the capacity to generate reappraisals of stressful events (Joormann and Gotlib 2010; Malooly et al. 2013; Pe et al. 2013b; Rowland et al. 2013; Weber et al. 2014). More specifically, generating reappraisals of adverse situations requires the ability to inhibit the (prepotent) negative aspect of the situation and to switch (shift focus) between negative and neutral mental sets when processing and re-interpreting the meaning of the situation (Malooly et al. 2013). In line with this, the left ventrolateral prefrontal cortex has been linked with the cognitive control of memory. It has been suggested that it is implicated in control processes guiding access to relevant information from semantic memory, particularly during conditions requiring goal-directed access to semantic knowledge. In addition, it may be implicated in post-retrieval selection processes that resolve competition between simultaneous active representations, that is, processes that select representations that are relevant to the current task over currently irrelevant or inappropriate associated information (that may be retrieved automatically). Functionality of these processes is also relevant for effective cognitive switching. A number of brain imaging studies have observed activation in left ventrolateral prefrontal cortex during tasks relying on these functions (for review see Badre and Wagner 2007).

However, whereas these executive functions clearly fit the demands during the generation of cognitive reappraisals, they may likewise fit the demands during the creative idea generation task. Thus, as individual differences in the capacity for cognitive reappraisal were also explained by unique variance of EEG alpha asymmetry during the generation of reappraisals that was independent from variance of EEG alpha asymmetry during the creative idea generation task, some additional demands must be implicated in cognitive reappraisal. It seems as if the importance of lateral asymmetry of activation in prefrontal cortex is enhanced when cognitive processes that are also active in novel idea generation are operating in an emotional context. Cognitive reappraisal requires the inhibition of the prepotent *emotional* aspect of a situation, and the ability to flexibly attend to and disengage from *emotional* aspects of a situation. Consequently, enhanced activity in the same areas or the additional recruitment of adjacent areas may be necessary, which cannot be demonstrated separately because of the coarse spatial resolution of the EEG.

The association of relevant executive processes with the asymmetry observed at the frontopolar electrodes is less obvious. The frontopolar electrode positions are located over the rostral limit of the superior frontal gyrus (Homan et al. 1987), which is part of a region that is often referred to as the “lateral orbitofrontal cortex”. Assumed functions of the rostral / lateral orbitofrontal region include the alteration and updating of the affective value and motivational relevance of a situation (Bechara et al. 2000; Kringelbach and Rolls 2004; Ochsner et al. 2004), demands that were explicitly required in the cognitive reappraisal task (but not in the creative idea generation task) of the present study.

Note that the so far discussed effects are correlations with individual differences in participants’ inventiveness in generating cognitive reappraisals. These correlations do not just reflect the effect of the condition itself. Consequently, it can be excluded that these findings may simply be attributed to the experience of anger. More angry states have consistently been linked to more left-lateralized activation in the dorsolateral prefrontal region (see Harmon-Jones 2004; Harmon-Jones et al. 2010). It would be grotesque to assume that individuals higher on the capacity for cognitive reappraisal (which showed relatively greater activation in the left hemisphere during the reappraisal task) would have experienced higher levels of anger. Nevertheless, as soon as the Reappraisal Inventiveness Test will have been extended to other emotions, a replication study using vignettes inducing other emotional states is certainly warranted.

As for task effects irrespective of individual differences in the capacity for generating cognitive reappraisals, no significant average effects of task condition on EEG alpha asymmetry at the ventrolateral or frontopolar electrode positions were observed when the cognitive reappraisal task was compared to the creative idea generation task without emotional component. This indicates that inter-individual differences in EEG alpha asymmetry changes were greater than the average effect of the task condition. Obviously not all participants, but only those higher on the capacity for cognitive reappraisal, showed more left-lateralized activation of the ventrolateral prefrontal and the frontopolar cortex during the generation of cognitive reappraisals over and above the activation observed during the generation of novel ideas without emotional component. The laterality (EEG alpha asymmetry) approach used in the present study seems to be particularly sensitive for these inter-individual differences in unique activations.

The small and marginally significant average effect of the reappraisal condition in comparison to the creative idea generation task at the dorsolateral prefrontal electrode positions may reflect the experience of anger, which was induced by the vignettes of the Reappraisal Inventiveness Test. Whereas the ventrolateral prefrontal cortex is involved in top-down modulation of affective responses, the dorsolateral portion seems to be more involved in the bottom-up part of the process and thus, in the actual experience of current affective states, as well as in the modulation of behavioral responses in the context of affective processing (Davidson 2004b; Johnstone et al. 2007; Ochsner and Gross 2007). The experience of anger, and accompanied approach motivation, has consistently been linked to relative left-lateralized activation in the dorsolateral prefrontal cortex (Harmon-Jones 2004; Harmon-Jones et al. 2010).

An average shift to the left at the ventrolateral prefrontal sites was observed during both tasks compared to the resting condition, which is in accordance with activations of ventrolateral prefrontal cortex after instructions to use cognitive reappraisal during negative picture viewing (Dillon and Pizzagalli 2013; Kalisch 2009; Ochsner et al. 2002; Phan et al. 2005), as well as during creative idea generation (Benedek et al. 2014; Fink et al. 2009), which has often been more prominent in the left hemisphere.

A potential limitation of the present study is that the focus of the vignettes in the cognitive reappraisal task was exclusively on coping with anger-evoking events. While there is no obvious reason to expect that an individual’s capacity for generating cognitive reappraisals differs across emotions, this has not yet been empirically confirmed. Moreover, apart from its likely correlation with the regulation of other emotions, the capacity for effective coping with anger-evoking events as such is relevant to a variety of conditions. For instance, defective regulation of anger may contribute to the propensity for violent behavior, which is a major public health problem (Davidson et al. 2000; Harmon-Jones 2004; Nestor 2002). The sample was female only. Effects sizes are small, which, however, has to be expected in investigations of EEG correlates of psychological variables for natural reasons. Moreover, the behavioral test for the assessment of the capacity for reappraisal that was used in the present study had been tailored for a student population for research purposes (Weber et al. 2014). The findings should be replicated in other populations, for which the test can be easily adapted.

A further limitation is that the capacity for generating alternative appraisals of stressful events is an important but certainly not the only factor determining the success of efforts to cognitively reappraise adverse situations. In the field of psychotherapy, the use of maximum performance measures is a new concept, referring to individuals’ potential for flexibility and the range and quality of cognitive constructions of which they are capable rather than what they typically do. In their typical performance, individuals may rely on a few strategies for reappraisal that have become habitual and automatic over time. Possible alternatives for construing emotional experiences may no longer be consciously considered unless people are confronted with new situations, in which they cannot rely on their routinely strategies for reappraisal. For effective intrapersonal emotion-regulation a broad repertoire of different strategies and its flexible, situation-appropriate use is necessary. The fluency and flexibility aspects of maximum performance tests such as the Reappraisal Inventiveness Test can be used to quantify the reappraisal potential of individuals.

Hence, future studies are warranted to assess the value of the Reappraisal Inventiveness Test as a diagnostic tool. For example, it will be interesting to examine whether the ability to invent different reappraisals for anger inducing situations better prepares individuals to maintain their self-control when provoked or frustrated. Additionally, the Reappraissal Inventiveness Test may prove as valuable tool for the evaluation of the psychotherapeutic process by providing a quantitative measure of the reappraisal skills of a client by comparing the “old” reappraisals (i.e., those that they had retrieved from prior experiences) from those that are “new” (i.e., invented during the therapy process).

In conclusion, the present study demonstrated neural correlates of the capacity for cognitive reappraisal in terms of an individual’s inventiveness in generating alternative appraisals for negative emotional events. During cognitive reappraisal efforts, individuals higher on the capacity for generating cognitive reappraisals showed more relative left-lateralized activity in lateral prefrontal cortex, specifically in the ventral region extending toward the frontal pole. The findings indicate that individuals higher on the capacity for cognitive reappraisal are more capable or more prone to recruit appropriate brain regions when the situation demands coming up with alternative appraisals of stressful events.

There is ample evidence that deficits in emotion regulation skills, though not specifically in the ability to perform cognitive reappraisal, contribute to the development of depression and related psychopathology. Studies confirmed the trainability of cognitive reappraisal and showed that incorporating emotion regulation training can enhance the effectiveness of treatments (Berking et al. 2014; Denny and Ochsner 2014; Hofmann et al. 2012). However, not all interventions are equally promising for all patients. The identification of neural correlates of factors determining the capacity to effectively implement cognitive reappraisal for self-regulation of negative affect may assist in determining appropriately targeted intervention. When the capacity is impaired, for instance in older people on account of declines in relevant executive functions, training of other strategies such as distraction may be more effective (Smoski et al. 2014). Finally, the present findings provide a neurophysiological validation of the used behavioral test (Reappraisal Inventiveness Test), which may help to match patients to treatments targeting their individual deficits.

## Footnotes

1 Additional data were obtained for purposes related to other, non-overlapping research questions.

2 No significant correlations were observed between performance (number of generated ideas) in the Alternate Uses Task and EEG alpha asymmetry during either the reappraisal or the Alternate Uses Task.
